# Reduction of integrin alpha 4 activity through splice modulating antisense oligonucleotides

**DOI:** 10.1038/s41598-019-49385-6

**Published:** 2019-09-10

**Authors:** May T. Aung-Htut, Iain Comerford, Russell Johnsen, Kerrie Foyle, Sue Fletcher, Steve D. Wilton

**Affiliations:** 1 Centre for Molecular Medicine and Innovative Therapeutics, Murdoch University, Perth, Western Australia Australia; 2 Perron Institute for Neurological and Translational Science and The Centre for Neuromuscular and Neurological Disorders, The University of Western Australia, Nedlands, Western Australia Australia; 3 Chemokine Biology Lab, School of Biological Sciences, University of Adelaide, Adelaide, South Australia Australia

**Keywords:** RNA splicing, Molecular medicine

## Abstract

With recent approvals of antisense oligonucleotides as therapeutics, there is an increasing interest in expanding the application of these compounds to many other diseases. Our laboratory focuses on developing therapeutic splice modulating antisense oligonucleotides to treat diseases potentially amendable to intervention during pre-mRNA processing, and here we report the use of oligomers to down-regulate integrin alpha 4 protein levels. Over one hundred antisense oligonucleotides were designed to induce skipping of individual exons of the *ITGA4* transcript and thereby reducing protein expression. Integrin alpha 4-mediated activities were evaluated in human dermal fibroblasts and Jurkat cells, an immortalised human T lymphocyte cell line. Peptide conjugated phosphorodiamidate morpholino antisense oligomers targeting *ITGA4* were also assessed for their effect in delaying disease progression in the experimental autoimmune encephalomyelitis mouse model of multiple sclerosis. With the promising results in ameliorating disease progression, we are optimistic that the candidate oligomer may also be applicable to many other diseases associated with integrin alpha 4 mediated inflammation. This highly specific strategy to down-regulate protein expression through interfering with normal exon selection during pre-mRNA processing should be applicable to many other gene targets that undergo splicing during expression.

## Introduction

Since the first report of antisense oligonucleotides (AO) being used to block viral replication, by Zamecnik and Stephenson^1^, there was great optimism for their possible application as therapeutics. Indeed, depending on the AO chemistry and backbone, several mechanisms of antisense action have been identified, including splice modulation that has gained momentum over the last few years. The accelerated or full approval of nucleic acid drugs by the US Food and Drug Administration is an important achievement for the field^2^, and new drugs in the clinic include two splice modulating AOs; *Eteplirsen*^3^, an exon skipping phosphorodiamidate morpholino oligomer (PMO) for the treatment of a subset of Duchenne muscular dystrophy patients and *Spinraza*, an AO promoting exon inclusion for the treatment of Spinal muscular atrophy^4^. Additional splice modulating AOs are also under investigation as therapeutics for genetic diseases caused by splice mutation defects that should be amendable to correction^5^. Our laboratory focuses on developing splice modulating AOs for many different conditions, and here we show that application of AOs is not limited to rescuing gene expression, but can also down-regulate gene expression through targeted disruption of normal exon selection and mRNA maturation. We describe the development and evaluation of exon skipping AOs designed to induce a non-functional isoform and thereby reduce the activity of integrin alpha 4 protein (ITGA4), also known as very late antigen 4 α subunit or CD49d.

ITGA4 is an α subunit of integrin receptors and associates with the β 1 chain (CD29) in very late antigen 4 or the β 7 chain of lymphocyte Peyer patch adhesion molecule^6^. The very late antigen 4 expressed on T cells plays an important role in inflammation by facilitating T cell migration to the tissue through interactions with the vascular cell adhesion molecule 1 (VCAM-1) on endothelial cells^7^. A humanised monoclonal antibody targeting ITGA4, natalizumab, is an approved drug for treating multiple sclerosis and Crohn’s disease^8^. Patients treated with natalizumab over several years show reduced, or remain free of, annual relapses and exhibit improved cognitive performance^9^. There is no doubt that ITGA4 is a relevant target in the treatment of multiple sclerosis (MS), but the risk of developing progressive multifocal leukoencephalopathy (PML)^10^ and the incidence of neutralising anti-natalizumab antibody in up to 50% of the patients^11^ limits the long term effectiveness of the treatment in these patients. Therefore, alternative therapies for multiple sclerosis must be explored.

ATL1102, a 2′-*O*-(2-methoxyethyl) modified gapmer AO, designed to induce RNase H degradation of the *ITGA4* transcript and suppress ITGA4 expression, was developed by Myers and colleagues^12^, and a clinical trial with ATL1102 showed a reduced number of active lesions in patients with relapsing-remitting MS^13^. This work further validated antisense therapy as an alternative strategy for the treatment of MS with ITGA4 as a therapeutic target. However, the phosphorothioate backbone modifications incorporated into ATL1102 has been shown to elicit non-specific cellular responses, including platelet activation,^14^ and inflammatory responses in mouse^15^ and therefore long term use of ATL1102 is still in question. A DNAzyme, another class of nucleic acid therapeutic, mediated down-regulation of the *ITGA4* transcript was also explored^16^, however cleavage of *ITGA4* transcript by DNAzymes was only shown in cell-free assay and down-regulation of ITGA4 protein was not reported.

We hypothesise that inducing specific AO-mediated excision of either an in-frame, encoding a crucial domain, or an out-of-frame exon to disrupt the reading frame would disrupt production of functional ITGA4 and hence lower the activity of this gene product. After *in silico* analysis of the *ITGA4* transcript for predicted motifs involved in splicing, we designed AOs to induce skipping of individual exons from the *ITGA4* transcript. Exon skipping was induced for most exons targeted but with very variable efficiencies. Down-regulation of ITGA4 protein and activity were confirmed in healthy fibroblasts and Jurkat cells. *In vivo* validation of splice modulating AO-mediated down-regulation of ITGA4 activity was performed in the experimental autoimmune encephalomyelitis (EAE)^17^ mouse model of MS, by injecting peptide conjugated phosphorodiamidate morpholino oligomers (PPMOs) that were designed to induce specific *Itga4* exon skipping, into mice with chronic EAE. Half of the EAE mice treated with an *Itga4* exon 4 skipping PPMO showed a delay in disease progression, although the results were not statistically significant. We are optimistic that further refinement of the study, including optimization of the PPMO dosage regimen will confirm the therapeutic potential of the *Itga4* exon 4 skipping strategy.

## Results

### Analysis of the *ITGA4* transcript and structure

ITGA4 is a cell surface integrin receptor protein encoded by 28 exons (Supplementary Fig. S1). The functional full-length protein contains an extracellular domain encoded by exons 1–26, a transmembrane domain encoded by exon 27 and a cytoplasmic domain translated from exon 28. The reading frame of the *ITGA4* transcript is shown in Supplementary Fig. S1 and skipping individual exons 2, 3, 4, 5, 6, 7, 10, 11, 12, 13, 14, 15, 17, 18, 20 or 22 will disrupt the reading frame of the *ITGA4* transcript, resulting in an internally truncated mRNA that cannot be translated into a functional protein and may be degraded via nonsense-mediated decay. On the other hand, individual excision of exons 8, 9, 16, 19, 21, 23, 24, 25, 26 or 27 will maintain the open reading frame and a shorter but potentially semi-functional protein isoform may be translated from these transcripts. Exon 27 of ITGA4 encodes the transmembrane domain and removing this exon should generate a non-membrane bound ITGA4 protein that may act as a decoy in circulation or accumulate within the cells.

### Initial AO screen using healthy human dermal fibroblasts

**Figure 1:**
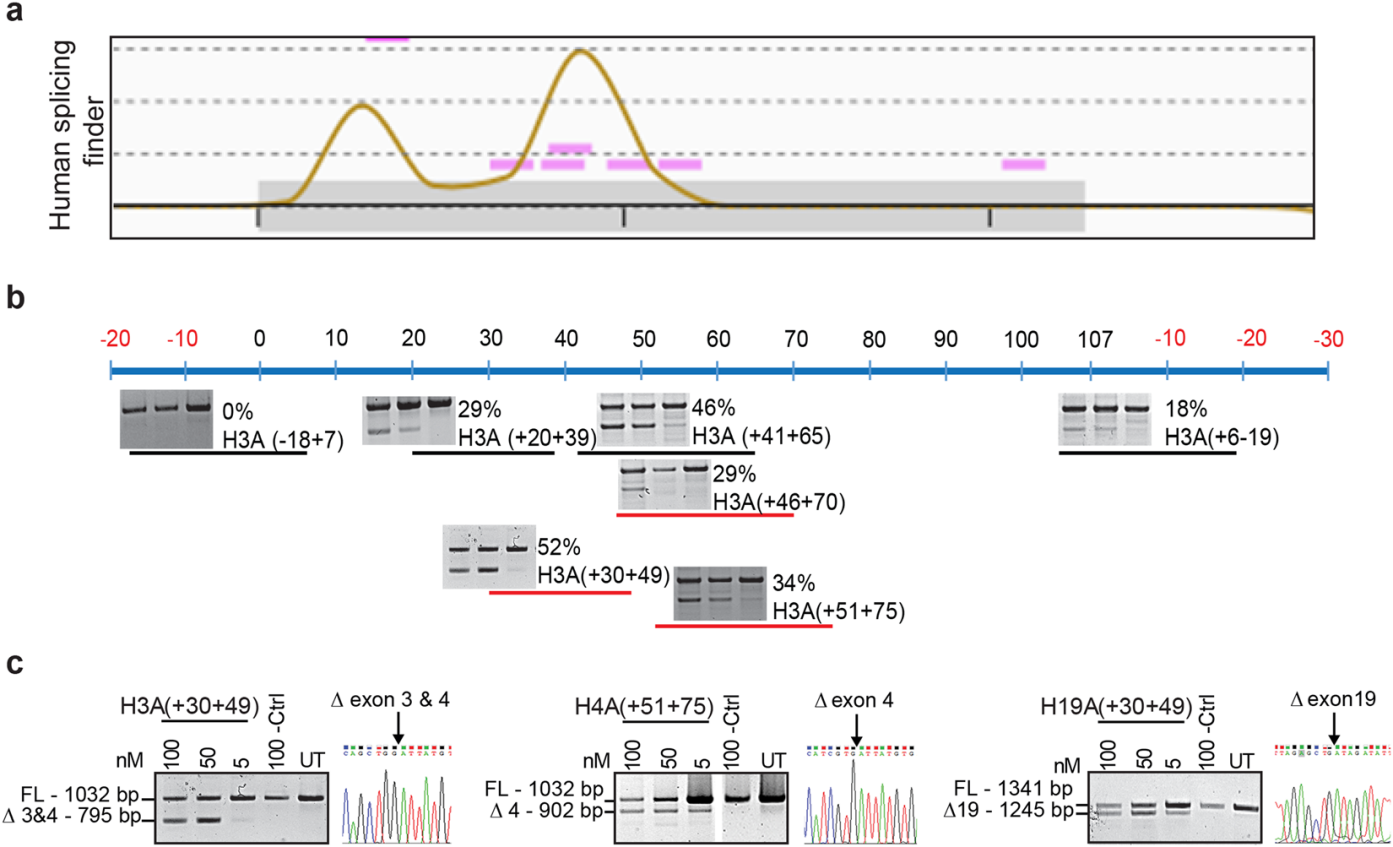
Design and experimental evaluation of *ITGA4*-targeting AOs. AOs designed to induce exon 3 skipping of the *ITGA4* transcript are shown. (**a**) Splice enhancer motifs predicted by the Human splicing finder tool for exon 3 (grey box) and partial intron 2 and 3 (solid black lines outside the grey box). The height of the peaks represents the collective strength of the motifs. Purple bars show the location and strength of each predicted motif. (**b**) AO designed for exon 3 based on the analysis in (**a**). The negative numbers in red indicate the nucleotide positions of the intron relative to exon and the numbers in black represent the nucleotide positions of the exon relative to the acceptor site. Short black lines represent AOs designed for initial screen that cover the sequences around the peaks, acceptor and donor sites. The short red lines represent microwalking AOs designed to refine AO H3A(+41 +65) to achieve maximum exon skipping efficiency with minimal AO length. Only selective microwalking AOs for H3A(+41 +65) are shown as examples for illustration. Gel fractionation of RT-PCR products of *ITGA4* amplicons from healthy fibroblasts transfected with 2OMe PS AOs at various concentrations (100, 50 and 5 nM from left to right) for 24 hr is shown above each AO. The percentage of exon skipping determined as described in Materials and Methods and the AO names are also shown. (**c**) The top three AOs for exon 3, 4 and 19 selected after microwalking to perform further studies. RT-PCR products of *ITGA4* amplicons from healthy fibroblasts after transfection with the corresponding 2OMe PS AOs at indicated dosages for 24 hr and confirmation of exon skipping by Sanger sequencing are shown. Ctrl: control AO, UT: untreated. The gels were cropped for presentation and full-size gels are presented in Supplementary Fig. S5.

Exon skipping AOs targeting exons 2–27 of the *ITGA4* transcript were designed after *in silico* analysis of splice motifs using Human splicing finder^18^ (Fig. 1). AOs (25–26 oligonucleotides long) with 2′-*O*-methyl base modifications (2OMe) on a phosphorothioate (PS) backbone were used for the initial study. All sequences tested in this study are shown in Supplementary Table S2. AOs were named according to our nomenclature previously described^19^ (Supplementary Fig. S1). These AOs were transfected into healthy human fibroblasts over a range of concentrations (0–100 nM) for 24 hours before RNA extraction for *ITGA4* transcript analysis by RT-PCR (Fig. 1b). Dose-dependent exon skipping in fibroblasts was induced by most AOs (4 out of 5 AO) designed to target the *ITGA4* transcript (Fig. 1b,c). An AO sequence that does not have 100% homology in the human genome and showed no effect on the *ITGA4* transcript after transfection (Supplementary Fig. S1) was used as a negative control AO for all fibroblast transfections (Fig. 1c).

Exon skipping efficiency after AO transfection at 100 nM was estimated according to the percentage of exon-skipped products, relative to total products [skipped product/(full-length + skipped product)]. AO efficacy was categorised as high (exon skipped product >30%), moderate (exon skipped product is 10–30%) and low (exon skipped product <10%) or no exon skipping (Supplementary Table S3). Ten out of 26 exons in the *ITGA4* transcript could be skipped with more than 30% efficiency, and there was a general trend for all frame-shifting exons being skipped at a lower level. Only two frame-shifting exons, exon 3 and 4, could be skipped with high efficiency, however, the AOs designed to target exon 3 caused skipping of both exon 3 and 4, resulting in an in-frame transcript (Fig. 1b,c).

Of all the AOs tested, three AOs induced more than 30% exon skipping; H3A(+41 +65) that caused skipping of both exons 3 and 4, H4A(51 + 75) and H19A(+30 +49) were selected for further optimisation of AO design to enhance exon skipping (Fig. 1b). A series of overlapping AOs targeting these three annealing sites were designed, transfected into the fibroblasts and exon skipping efficiencies assessed (Supplementary Table S4). Except for the shorter (20 mers) AOs, H3A(+30 +49) (Fig. 1b) that slightly increased exon skipping efficiency compared to the original sequence, and H3A(+41 +65), none of the new AOs showed improved exon skipping. Interestingly, exon 19 skipping was completely abolished when the annealing position was shifted a single base upstream or downstream from the original target site of H19A(+30 +49) (Supplementary Table S4).

**Figure 2:**
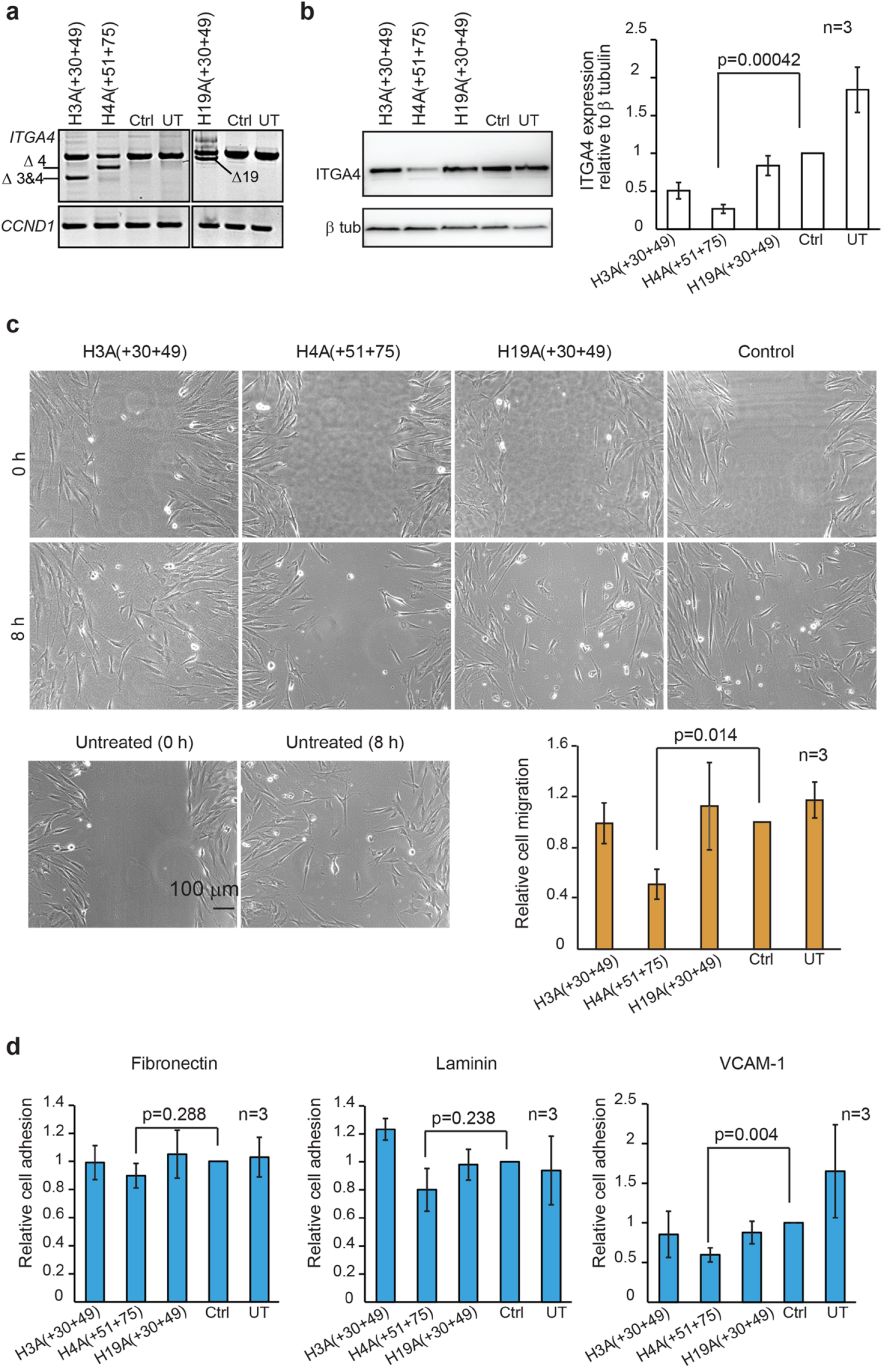
Evaluation of the best performing exon skipping 2OMe PS AOs in healthy dermal fibroblasts. The *ITGA4* transcript, ITGA4 protein expression and activity of healthy dermal fibroblasts after treatment with the top three exon skipping AOs targeting exon 3, 4 and 19 for 48 hr were analysed. (**a**) Gel fractionation of RT-PCR products of *ITGA4* amplicons amplified from healthy dermal fibroblasts transfected with the 2OMe PS AOs at 100 nM for 48 hr. Ctrl: control AO, UT: untreated. *CCND1* transcript encoding cyclin D protein was used as a loading control. Transcripts with exon(s) missing are labelled. (**b**) Western blot analysis of ITGA4 protein expression, (**c**) analysis of fibroblast migration using an established wound healing assay, and (**d**) relative fibroblast adhesion to extracellular matrix fibronectin, laminin and VCAM-1 using the treated and untreated healthy dermal fibroblasts from (**a**). Beta tubulin was used as a reference protein for western blot analysis. Densitometric analysis of western blots and relative migration and cell adhesion analysed as described in Materials and Methods for three biological replicates are shown as bar graphs. The gels and blots were cropped for presentation and full-size gels and blots are presented in Supplementary Fig. S5. Error bar; SEM. Scale bar 100 µm.

Based on these results, three 2OMe PS AOs; H3A(+30 +49), H4A(51 +75) and H19A(+30 +49) (Fig. 1c), were further evaluated for their effect on ITGA4 protein expression after confirmation of exon skipping using RT-PCR at 48 hours (Fig. 2a). The *CCND1* transcript encoding cyclin D1 protein was used as a loading control for the RT-PCR and we observed a reduction of the full-length *ITGA4* transcript in samples treated with H4A(51 + 75) and H19A(+30 +49). As shown in Fig. 2b, there was a decrease in ITGA4 protein expression after treatment of fibroblasts with H4A(51 + 75) for 48 hours, compared to the treatment with the control AO. In addition, fibroblasts treated with H4A(51 + 75) showed slower migration compared to the control AO treated fibroblasts in the wound healing assay (Fig. 2c). Biological replicates of RT-PCR, western blots and migration assays independently performed are shown in Supplementary Fig. S2. The interaction between ITGA4 and extracellular matrix proteins fibronectin, laminin and VCAM-1 was also assessed by measuring relative cell adhesion to the plates pre-coated with these matrixes, as shown in Fig. 2d. Compared to the control AO treated fibroblasts, fewer fibroblasts adhered to only the VCAM-1 coated plate after treatment with AO H4A(51 + 75). These results showed that among three AOs tested, H4A(51 + 75) treatment reduced not only ITGA4 protein expression, but also ITGA4 function as demonstrated by slower cell migration and interaction with VCAM-1.

### Validation of AOs targeting the *ITGA4* transcript in the T lymphocyte cell line, Jurkat

Fibroblasts served as a useful, convenient system to perform the initial AO screen and identify the most efficient exon skipping compounds. However, *ITGA4* is highly expressed and important for immune cell function, therefore the exon skipping AOs identified from the fibroblast screen were further verified in the T lymphocyte cell line, Jurkat. Five AO sequences, H3A(+41 +65), H3A(+30 +49), H4A(+51 +75), H19A(+30 +49) and H27A(+20 +44) were synthesized as PMOs, as we have shown that these compounds conferred more robust splice switching *in vitro* and *in vivo*^20,21^. The rationale behind choosing these particular five AOs was to (i) compare the efficacy of PMO of different lengths [H3A(+41 +65) vs H3A(+30 +49)], (ii) confirm reduction of the full-length *ITGA4* transcript, protein and activity observed after transfection with H4A(+51 +75), (iii) examine if modifying the AO backbone as a PMO could further increase the efficacy of H19A(+30 +49) in reducing ITGA4 protein levels and activity and (iv) ascertain if excision of the in-frame exon 27 would produce a soluble protein lacking the transmembrane domain encoded by that exon. The control sequence (GTC) designed and supplied by GeneTools^22^ was used as a negative control for all morpholino oligomer studies.

**Figure 3:**
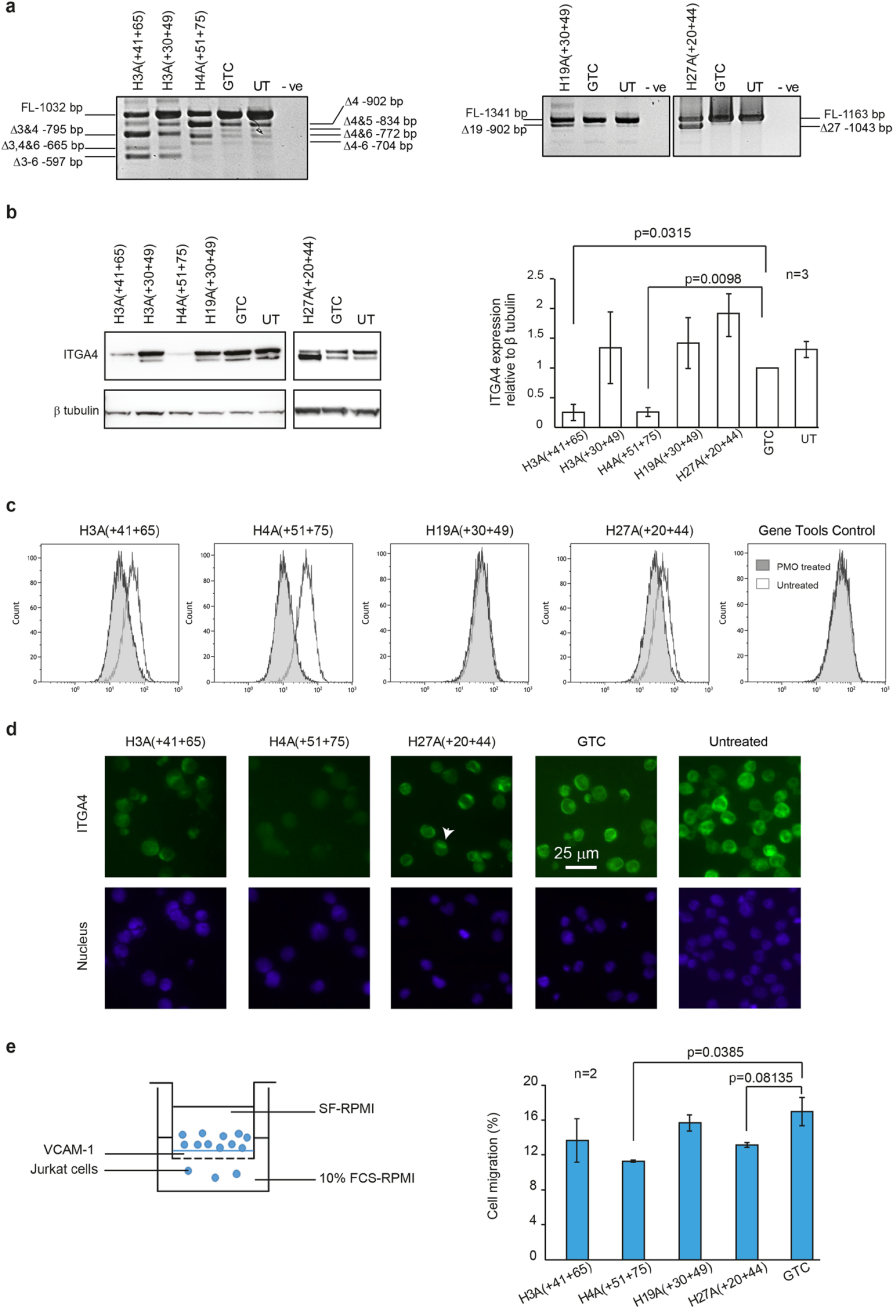
Evaluation of the best performing exon skipping PMOs in Jurkat cells. The *ITGA4* transcript, ITGA4 protein expression and activity in Jurkat cells treated with indicated PMOs for three days were analysed. (**a**) Gel fractionation of RT-PCR products of the *ITGA4* amplicons and (**b**) western blot analysis of ITGA4 protein from Jurkat cells nucleofected with PMOs, as indicated above the gel, at 50 µM for three days. Beta tubulin was used as a reference protein. Densitometric analysis of three biological replicates for western blots is shown on the right. Error bar; SEM. (**c**) Flow cytometry analysis of surface receptor ITGA4 on Jurkat cells treated with 50 µM of PMO for three days. (**d**) Immunolabeling of ITGA4 protein in Jurkat cells treated with 50 µM of PMO for three days. Arrow head shows cell with intracellular accumulation of ITGA4 protein. Refer to Supplementary Fig. S3 for the display lookup tables. Green: ITGA4, blue: nucleus. Scale bar 25 µm. (**e**) Migration of Jurkat cells, treated with 50 µM of indicated PMO for three days, via interaction with VCAM-1, was assessed as shown in the illustration on the left and the percentages of cells migrated after 5 hr are shown as a bar graph. The experiment was performed in duplicate. Error bar; SD. GTC: Gene Tools control AO, UT: untreated. The gels and blots were cropped for presentation and full-size gels and blots are presented in Supplementary Fig. S5.

All PMOs were nucleofected into Jurkat cells and the *ITGA4* transcript and protein expression were analysed. High micromolar concentrations of PMOs were tolerated in cells compared to 2OMe AOs where cell death was observed even at the lower concentrations, when delivered as cationic lipoplexes (data not shown). Similar to observations in transfected fibroblasts, both exons 3 and 4 of the *ITGA4* transcript were skipped in Jurkat cells treated with PMO H3A(+41 +65) or H3A(+30 +49) (Fig. 3a). However, PMO H3A(+41 +65) induced a much higher percentage of exon skipping than H3A(+30 +49). Two additional *ITGA4* transcripts missing exons 3 to 6, or exon 3, 4 and 6 together were also observed after both treatments. The PMO targeting exon 4, H4A(+51 +75) induced predominantly exon 4 skipping and additional transcripts missing either exons 4, 6, or 4, 5 and 6 (Fig. 3a). In addition to the full-length *ITGA4* amplicon, low levels of four splice isoforms missing exons 4, exon 4 + 5, exon 4 and 6, or exon 4 to 6 were observed in both untreated and GTC PMO treated Jurkat cells. PMO H19A(+30 +49) induced less than 10% exon 19 skipping, however, exon 27 was excised in approximately 50% of the total *ITGA4* transcript in cells treated with PMO H27A(+20 +44) (Fig. 3a).

When ITGA4 protein expression was analysed in PMO-treated Jurkat cells 3 days after administration, the full-length ITGA4 protein was decreased in PMO H3A(+41 +65) and H4A(+51 +75) treated cells, compared to those transfected with GTC PMO (Fig. 3b). Protein reduction was maintained up to 6 days in the actively growing Jurkat cells (Supplementary Fig. S3), while no significant changes in ITGA4 protein level were observed in cells after PMO H3A(+30 +49) or H19A(+30 +49) treatment. Interestingly, an increase in a truncated ITGA4 isoform that co-migrated with ITGA4 precursor protein (the lower band), was observed in Jurkat cells treated with PMO H27A(+20 +44). Biological replicates of RT-PCR and western blots independently performed are shown in Supplementary Fig. S3.

To complement western blot analysis, flow cytometry analysis of ITGA4 receptor on the Jurkat cells treated with PMOs was also performed (Fig. 3c). PMO 3A(+30 +49) treated cells were not included in this analysis since no changes in ITGA4 protein expression was observed after western blotting. Similar to the western blot analysis, reduction of ITGA4 surface receptor protein was observed for PMO H3A(+41 +65) and H4A(+51 +75) treatment, but not in H19A(+30 +49) or GTC treated Jurkat cells (Fig. 3c). In addition, we also analysed the location of ITGA4 protein in the PMO treated Jurkat cells shown to have a reduced level of ITGA4 protein by immunolabeling (Fig. 3d). ITGA4 protein was localised both on the cell membrane and in the cytoplasm of GTC treated and untreated cells. Reduction of both cell membrane and cytoplasmic ITGA4 protein was observed in H3A(+41 +65) or H4A(+51 +75) treated Jurkat cells. Interestingly, Jurkat cells treated with PMO H27A(+20 +44) showed a reduced level of ITGA4 on the cell membrane and accumulation of ITGA4 in the cytoplasm in some cells (refer to Supplementary Fig. S3 for the display lookup tables).

Next, the biological activity of ITGA4 and interaction with VCAM-1 was analysed using a cell migration assay (Fig. 3e). Jurkat cells treated with different PMOs were allowed to migrate through VCAM-1 coated transwell inserts, and the percentages of cells that migrated through the inserts after 5 hours were analysed and compared with those of the PMO GTC treated cells. PMOs H4A(+51 +75) or H27A(+20 +44) caused consistently slower Jurkat cell migration compared to the GTC PMO.

### *In vivo* validation of *ITGA4* exon skipping PPMOs in the EAE mouse model

Since *in vitro* assessment of exon skipping AOs targeting the *ITGA4* transcript showed significant down-regulation of the *ITGA4* transcript, protein and biological activity in both fibroblasts and Jurkat cells, we performed a proof-of-concept study *in vivo* using an EAE mouse model of MS. Murine *Itga4* transcript-specific AOs, with 2OMe modifications on a PS backbone, targeting exon 3, 4 and 27 were designed and assessed for exon skipping efficiency using the murine myoblast cell line, *H2k mdx*^23^. The target exons selected were determined by our study in human fibroblasts and Jurkat cells, and the mouse *Itga4* targeting sequences are shown in Supplementary Table S2.

**Figure 4:**
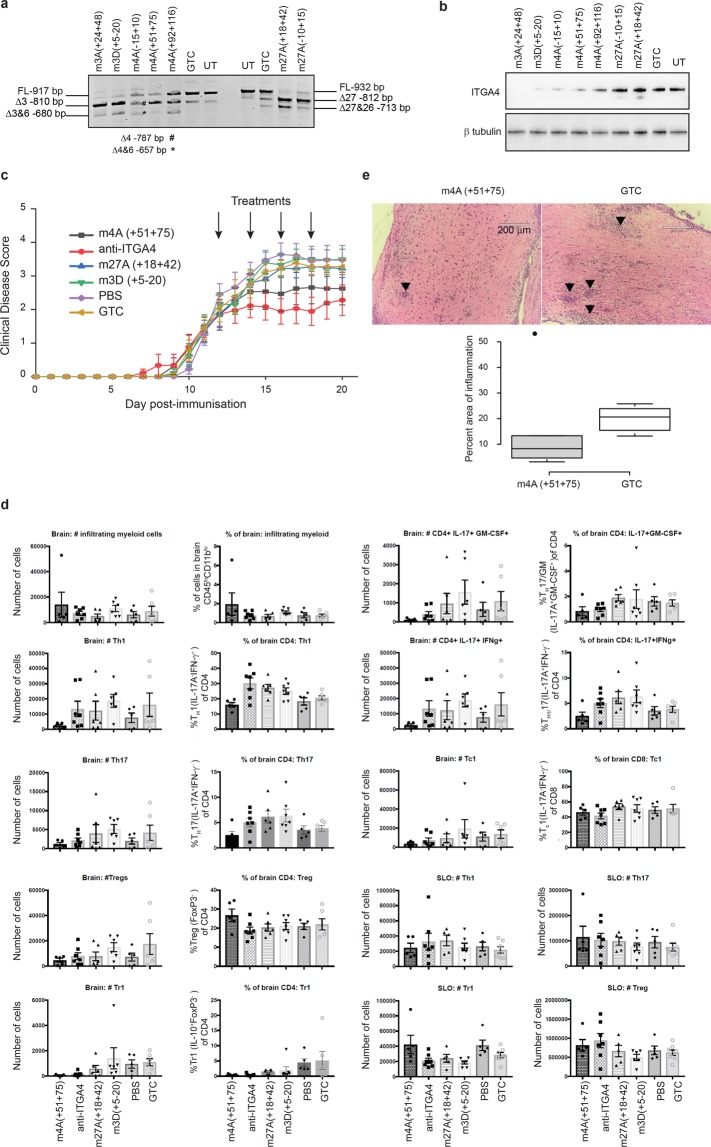
*In vivo* validation of peptide-conjugated PMOs (PPMOs) in the mouse EAE model of MS. (**a**) Gel fractionation of RT-PCR products of murine *Itga4* amplicons and (**b**) western blot analysis of ITGA4 protein expression from primary murine splenocytes nucleofected with PMOs, as indicated, at 50 µM for 48 hr. Beta tubulin was used as a reference protein. The transcripts with missing exons missing are shown. GTC: Gene Tools control AO, UT: untreated. (**c**) The disease course of EAE mice intraperitoneally injected with PPMOs targeting *Itga4* exons 3, 4 and 27 at 10 mg/kg on alternate days, as indicated by arrows. The clinical scores were determined over 21 days as described in Supplementary Table S5. (**d**) Flow cytometry analysis of infiltrating myeloid cells, and subsets of T cells in brain and secondary lymphoid organs (SLO: spleen/Inguinal lymph nodes pooled). ●: m4A(+51 +75) treated mice, ■: ant-ITGA4 antibody treatment, ▲: m27A(+18 +42) treatment, ▼: m3D(+5 −20) treatment, ◆: PBS treatment and ○: GTC treatment. Raw data is provided in Supplementary Tables S6 and S7. (**e**) Representative haematoxylin and eosin staining of the spinal cords isolated from EAE mouse treated with m4A(+51 +75) or GTC PMO (top panel). A box plot for the percentage of area in spinal cords, marked by inflammation (arrow heads), observed in m4A(+51 +75) and GTC treated groups (n = 5 per group). Scale bar: 200 µm. Error bars: SEM. GTC: Gene Tools control AO. The gels and blots were cropped for presentation and full-size gels and blots are presented in Supplementary Fig. S5.

Seven AO sequences were chosen for further study and synthesised as PMOs. The effects of the PMOs on exon selection and ITGA4 protein downregulation were confirmed in freshly isolated murine primary splenocytes after nucleofection, as shown in Fig. 4a,b. Two PMOs targeting exon 3 and one PMO targeting exon 4, m4A(+51 +75), showed nearly 90% decrease in both full-length *Itga4* amplicon and protein. PMOs targeting exon 27 induced efficient exon 27, and 27 and 26 skipping (nearly 100%). However, apart from a faint truncated protein band (~30kD smaller than the full-length protein), no significant changes were observed in the amounts of the full-length murine ITGA4 protein. Data from RT-PCR and western blot analysis of biological replicates are shown in Supplementary Fig. S4.

Peptide conjugated PMOs, m3D(+5–20), m4A(+51 +75) and m27A(+18 +42) were selected for further evaluation in the EAE mouse model. Rat anti-mouse ITGA4 antibody was used as a positive control, and the GTC PMO and PBS treatments were included as negative controls. Mice were assigned to experimental groups at disease onset and treated from day 12 post-immunisation. A statistically significant reduction in clinical disease score was observed in the anti-ITGA4 treated group compared to the PBS-treated group between days 16–20 post-immunisation (Fig. 4c). On average, the PBS-treated mice achieved a mean clinical disease score 2.5 points higher than that attained on the day treatment started, while the anti-ITGA4-treated cohort achieved a mean clinical disease score only around 0.75 points higher than that attained on the day treatment started. There was a trend for reduced disease manifestation in the PPMO m4A(+51 +75) treated group, with 50% of mice showing no disease progression after treatment. However, the data did not reach statistical significance as assessed by 2-way ANOVA analysis. None of the other PMO-treated groups of mice showed statistically significant changes in disease scores compared to the GTC or PBS treated mice.

The frequency of infiltrating myeloid cells, Th1, Th17, Treg, GM-CSF + Th17, Th1/17 (IL-17+ and IFNg+), Tr1 and Tc1 cells in the brain were assessed both in terms of percentage and absolute number (Fig. 4d and Supplementary Tables S6 and S7). These same subsets of T cells have also been assessed in secondary lymphoid organs (spleen/Inguinal lymph nodes pooled) to determine whether T cell priming was modified by any of the treatments. The average number of infiltrating myeloid cells are similar for all treatments, including the positive control treatment. There was a trend for lower numbers of Th1 and Th17 cells in the brain after treatment with PPMO m4A(+51 +75), however this was not statistically significant.

Longitudinal sections of frozen spinal cord from m4A(+51 +75) and GTC treated groups were stained using a standard haematoxylin and eosin protocol and analysed for the area of inflammation marked by infiltrating mononuclear cells within the tissue, as described in methods (Fig. 4e). As shown in Fig. 4e, the spinal cord section of the GTC treated mouse was highly inflamed with many infiltrated mononuclear cells while that of the m4A(+51 +75) treated mouse had fewer infiltrating mononuclear cells, indicating a lower level of inflammation. The infiltrating mononuclear cells were often present as active aggregates of inflammatory activity and also as diffuse activity in some areas. There was no inflammation detected in accompanying spinal nerve roots. The box plot in Fig. 4e shows the percent area of inflammation in spinal cords isolated from m4A(+51 +75) and GTC treated mice (five mice per group). There was one outlier mouse in the m4A(+51 +75) treated group that had a very high level of inflammation compared to the other mice in the same group. Apart from that particular mouse, the m4A(+51 +75) treated mice showed lower levels of inflammation compared to the GTC treated group.

## Discussion

Natalizumab is one of the approved treatments for patients with relapsing remitting MS^8^. The approval of natalizumab, despite the risk of developing fatal PML, showed that the therapeutic benefits of blocking ITGA4 outweigh the risks and ITGA4 is a validated and legitimate target for MS. However, there is speculation that the use of natalizumab, an IgG4 antibody, may have a Fab-arm exchange with naturally occurring human IgG4^24^, creating bispecific antibodies. This will have implications for the transport/replication of John Cunningham virus responsible for development of PML^25,26^. Therefore, other small molecule inhibitors^26^ of ITGA4 activity including RNaseH dependent AO^13^ and DNAzyme^16^ have been explored as alternative therapies for MS. Since splice modulation AOs are coming of age with the recent approvals of *Exondys* 51 and *Spinraza*, we report splice modifying AOs that reduce ITGA4 activity, identified by *in vitro* screening and validated *in vivo* in a mouse model of MS.

Since our aim is to introduce exon skipping as one strategy to downregulate ITGA4 activity, we confirmed exon skipping by RT-PCR before proceeding to protein expression and activity analysis. Although RT-PCR is semi-quantitative, unlike other quantitive analysis such as qPCR, RT-PCR provides us with information on changes in multiple transcript isoforms after treating with AOs (Fig. 3a). We observed an interesting trend in exon “skippability” of the *ITGA4* transcript during processing in that most in-frame exons tended to be more readily excised than out-of-frame exons. The AOs targeting exon 3 induced *ITGA4* transcripts missing not only exon 3 from the mature mRNA, but also exons 3 and 4. However, AOs targeting *ITGA4* exon 4 only caused exon 4 skipping, indicating that the presence of exon 3 is crucial for recognition of exon 4 during *ITGA4* pre-mRNA splicing and reflects a non-sequential order of exon splicing. This may also be due to the spliceosome recognising downstream splice sites when exon 3 is blocked, resulting in multiple exon skipping. We have observed a similar trend for inducing dystrophin exons 8 + 9 removal upon targeting exon 8 with a variety of AOs. In contrast, dystrophin exon 9 removal was specific when only that exon was targeted with an AO^27^. It is also possible that the short *ITGA4* intron 3 (92 bases) flanked by two larger introns (intron 2; 16,642 bases and intron 4; 3,468 bases) may have contributed to the dual exon splicing in the presence of AOs targeting exon 3. A similar arrangement is seen in dystrophin where intron 8 (1,113 bases) is flanked by large introns 7 (110,199 bp) bases and 9 (52,717 bases).

Although 2OMe PS AOs are cost effective for identifying motifs involved in pre-mRNA processing in the initial screening, it has been previously reported that the PS backbone causes detrimental cellular events including up- and down-regulation of multiple cellular pathways and non-specific splice modifications^28^. Therefore, we must confirm the effectiveness of the chosen AOs using a more specific, reliable and effective chemistry such as PMO. Generally we have found a good correlation with 2OMe AOs and PMO design of exon skipping sequences, where a strong 2OMe AO skipper is even more effective when evaluated as a PMO and a weak 2OMe AO most often translates to a poorly performing compound as a PMO^29^. The efficiency of exon skipping sequences was generally enhanced and more sustained, with reduced toxicity when the PMO compounds were used rather than 2OMe PS AOs. However, during optimization of some of the anti-*ITGA4* AOs, not all the outcomes from 2OMe AO transfection were directly translatable to PMO sequences (eg. exon 19 skipping AO). The reason for this is unclear but may reflect differences in the flexibility of the phosphorothioate and morpholino backbones and/or some other differences between the two chemistries. We have also compared the efficacy of PMOs of different lengths and found that the 20 mer PMO H3A(+30 +49) did not perform as well as 25 mer H3A(+41 +65).

Our attempt to create an ITGA4 protein isoform missing the transmembrane domain, encoded by exon 27, produced an interesting result. Exon 27 of both *Homo sapiens ITGA4* and murine *Itga4* transcripts are only 120 bases long and encodes 40 amino acids with an estimated molecular weight of 4.66 kD. Such a small difference in molecular weights of the intact and exon 27 deletion isoforms would be difficult to detect on a denaturing polyacrylamide gel since the glycosylated ITGA4 is migrating around 150 kD. We assumed that the both ITGA4 isoforms, with or without exon 27, would be migrating at a similar rate. However, we did observe a smaller band with a molecular weight of approximately 120 kD, similar to that expected for un-glycosylated ITGA4 protein (115 kD). This shorter isoform was present in both Jurkat cells and murine splenocytes treated with exon 27 skipping AO. For this observation, we speculate that excising the domain encoded by exon 27 may have somehow affected ITGA4 post-translational modification, being more pronounced in Jurkat cells than murine splenocytes. We also observed loss of ITGA4 protein from the cell membrane and intracellular accumulation in Jurkat cells treated with exon 27 skipping AOs. The exact mechanism by which most glycosylated protein was converted to unmodified protein requires further investigation and is beyond the scope of this report.

We also noticed that the percentage of reduction observed for the full-length *ITGA4* transcript did not fully reflect the percentage of ITGA4 protein reduction. This is because although transcription and translation processes are generally coupled, the relative amount of mRNA transcript to protein does not necessarily have to be linked. Multiple mechanisms may account for discrepancies between mRNA and protein levels.

Since we observed a consistent decrease of ITGA4 protein after treatment with PMO, we performed a proof-of-concept study to assess three exon skipping PPMOs in the EAE mouse model of MS. Although the *in vivo* data obtained did not reach statistically significant levels that might be expected to be therapeutic, the exon 4 skipping PPMO showed a clear trend towards delaying disease progression, similar to that resulting from the positive control anti-ITGA4 treatment. These results suggest that increased and/or more frequent dosing of PPMOs may confer a therapeutic benefit in EAE, but this remains to be confirmed. Furthermore, prophylactic (preventive) treatment in EAE, or use of a relapsing-remitting model of EAE, may be more amenable to PPMO intervention.

One of the concerns for any antisense therapy is the safety of the chemical modifications introduced to the nucleic acids to provide enhanced stability and specificity. Having a neutral backbone poses a limitation on cellular uptake, however PMOs are known to have an excellent safety profile, evident from the treatment of Duchenne muscular dystrophy patients with *Exondys 51*^30^ for more than eight years. In contrast, the negatively charged PS backbone incorporated in RNaseH dependent AOs are known to non-specifically bind serum proteins and induce inflammatory responses *in vivo*^15,31^. Therefore, compared to the RNasH dependent antisense drug ATL1102, the PMO identified in our study is anticipated to have a better safety profile. One of the challenges for PMOs is the poor uptake by cells, but this limitation is currently being addressed with the ongoing development of cell penetrating peptides and next generation morpholino chemistries.

Although the therapeutic potential of exon skipping PPMOs was assessed in the EAE mouse model, these compounds may be applicable to other pathological conditions that require reduction of ITGA4-mediated inflammation. Pinto-Mariz *et al*. showed that ITGA4 can be used as a prognostic marker and also may be targeted to reduce the muscle inflammatory response seen in Duchenne muscular dystrophy patients^32^. In addition, ITGA4 has been shown to be dysregulated in many cancer types and hence may be a potential target for therapy, assuming sufficiently effective splice switching/suppression can be induced. In conclusion, the antisense oligonucleotide designed to induce exon 4 excision from the mature *ITGA4* transcript has the potential as a therapeutic for many diseases involving inflammation. Downregulation of gene expression as a result of alternative splicing to generate non-functional transcript isoforms is an endogenous, commonly employed strategy for many genes, in particular many splicing factors such as SRSF2^33^ and hnRNPD^34^. Here, we show manipulation of exon selection using splice modulating AOs can offer new therapeutic avenues for serious human diseases.

## Materials and Methods

Refer to supplementary file for a detailed methods.

### Mutation-independent AO design to downregulate gene expression

Exon skipping AOs were designed after *in silico* analysis of splice motifs using Human splicing finder^18^ and SpliceAid^35^. 2OMe PS AO were either synthesized in-house on an Expedite 8909 or ordered from TriLink BioTechnologies. PMOs were from GeneTools LLC and peptide-conjugated PMOs (PPMO) were supplied by Sarepta Therapeutics.

### Cell propagation and transfection/nucleofection

Dermal fibroblasts were prepared from a skin biopsy of a healthy volunteer, with an informed consent (Murdoch University Ethics Approval #2013/156). All methods were performed in accordance with the relevant guidelines and regulations. Dermal fibroblasts were propagated in Dulbecco’s modified Eagle’s media (DMEM) supplemented with GlutaMAX™ and 10% fetal bovine serum (FBS). Cells were transfected with 2OMe PS AOs using Lipofectin® transfection reagent and RNA was extracted using Trizol after 24 or 48 hr. Protein analysis was performed at 48 hr. Jurkat cells were supplied by the European Collection of Cell Cultures (ECACC; Salisbury, United Kingdom) as catalogue number 88042803, and purchased from CellBank Australia and maintained in 10% FBS, RPMI-1640. The PMOs were nucleofected into Jurkat cells using the P3 Primary Cell 4D-nucleofactor X Kit (Lonza) at 50 µM using program CL-120. RNA and protein expression were analysed on day 3.

### RT-PCR assays

RT-PCR was performed using a Superscript III One-Step RT-PCR kit (Thermo Fisher) using 50–100 ng of total RNA to amplify *ITGA4* transcript. Primers and cycling conditions are detailed in Supplementary Tables S1. The images were captured using auto exposure setting and Fusion FX system (Vilber Lourmat, Marne-la-Vallée, France). Image processing was performed for the entire image. Product identity was confirmed by band purification and DNA sequencing at the Australian Genome Research Facility, Perth, Western Australia.

### Western blotting

Western blot analyses of ITGA4 and beta tubulin proteins were performed on approximately 20 µg of protein using anti-ITGA4 antibody (Cell Signaling Technology, cat. no. 4600)^36^ at 1:1000 dilution and rabbit polyclonal anti-β-tubulin (Invitrogen™, cat. no. PA1-41331)^37^ at 1:1000 dilution overnight at 4 °C. Polyclonal goat anti-rabbit immunoglobulins/HRP (Dako, cat. no P0448)^38^ at a dilution of 1:10,000 and Luminata Crescendo western HRP substrate were used for immunodetection. The blots were exposed for a serial scan of 30 s using the Fusion FX system (Vilber Lourmat, Marne-la-Vallée, France) and the entire image was processed. Quantification was performed using Image J (Rasband, W. S., ImageJ, U. S. National Institutes of Health, Bethesda, Maryland, USA).

### Wound healing assay

The assay was performed 48 hr after transfection with 2OMe PS AOs as described^39^ with slight modifications. A ‘wound’ was created by scraping the monolayer of transfected fibroblasts with a pipet tip. Images of the wound were captured at 10X magnification using a Nikon TS100 microscope and NIS-Elements software at 0 and 8 hours. The dimension of the wound gap at 0 and 8 hour images was assessed using Image J. The average cell migration of 3 biological replicates was calculated. Data were normalised to that obtained from control AO treated fibroblasts.

### Cell adhesion assay

The assay was performed as described^40^ with slight modifications. Cell suspension (50 µl) labelled with 2 μM calcein AM fluorescent dye was added to each well coated with different matrixes and incubated at 37 °C for 30–40 min. The microplates were washed four times with PBS, and the remaining adherent cells were measured using a Beckman Coulter DTX-880 Multimode Detector plate reader with excitation and emission wavelengths of 488 nm and 512 nm, respectively. The fluorescent signals from total cells were analysed in a separate microplate, omitting the wash steps. Background signals were subtracted from all samples and the percentages of adhered cells were calculated. The results were normalised to the sample treated with the control AO.

### Jurkat cell migration assay

The assay was performed as described^41^ with slight modifications. Jurkat cells nucleofected with PMO for 48 hr were added into the upper-sides of transwell migration inserts coated with VCAM-1 and allowed to migrate to the lower chambers for 5 h at 37 °C. Cells from both chambers were collected and incubated with 2 μM calcein AM fluorescent dye for 30 min before measuring fluorescent signals as described above. The percentages of cells that migrated to the lower chambers were calculated and normalised to the signals generated by the cells treated with GTC PMO.

### *In vivo* validation in EAE mouse model

*In vivo* validation of PPMO was performed at the University of Adelaide (S-2017-092) and  approved by the University of Adelaide ethics committee. All methods were performed in accordance with the relevant guidelines and regulations. Sixty female C57Bl/6 J mice (7–12 weeks) were obtained from the Animal Resources Centre in Perth. Mice were housed in individually ventilated cages with access to food and water *ad libitum*. Mice were immunised with 200 µg of MOG35-55 (BioNovus Life Science) peptide emulsified 1:1 in complete Freund’s adjuvant (Sigma) in a total volume of 100 μl per mouse, subcutaneously in the hind flank. On day 0 and on day 2, mice received 300 ng of pertussis toxin (Sigma) in a total volume of 250 μl PBS intravenously. Mice were weighed and clinical disease assessed daily in a blinded manner according to the criteria described in Tables S5. Eight of the 60 mice immunised were not assigned to experimental groups because they had either already met euthanasia criteria or developed atypical EAE characterized by ataxia and loss of co-ordination that cannot be adequately scored using the standard EAE clinical disease assessment. Mice that were found dead or were culled due to meeting animal welfare euthanasia criteria ( > 20% weight loss or excessive clinical disease) were scored at the same clinical score they had last attained for the remainder of the study.

Mice were intraperitoneally treated with 10 mg/kg of PPMO in PBS on day 12 and then every 48 hr thereafter. For rat anti-mouse ITGA4 antibody (Cat. 1520-14, Assay Matrix Pty Ltd) treatment as a positive control, mice were given 25 mg/kg on day 12 and 5 mg/kg every 48 hr thereafter. Equivalent volumes of PBS were administered intraperitoneally to control mice at the same time.

### Histology

The lumbar part of spinal cords embedded in OCT and snap frozen were sectioned on a cryostat (Leica CM1900), and mounted on microscope slides. Sections were stained using a standard haematoxylin and eosin stain^42^ for microscopic identification of inflammation using a Nikon Eclipse 80i microscope at 10X magnification using NIS-Elements software. Using NIH ImageJ software, the total area of each tissue section and areas of inflammation, with the presence of mononuclear inflammatory cells within the tissue were selected and measured. For each microscopic image analysed, measurements of areas of inflammation were presented as percent of total area of the field of views analysed. The operator was blinded for analysis.

### Immunolabeling

For immunolabeling, Jurkat cells were fixed in ice-cold Acetone:MetOH (1:1) for 5 min before incubation with primary antibody (Cell Signaling Technology, cat. no. 8440) diluted in TBST with 1% goat serum at 1:200 for 1 h at room temperature. The coverslips were then washed three times with TBST before incubation with Alexa 488 labelled goat anti-rabbit secondary antibody (ThermoFisher cat. no. A27034) diluted in TBST with 1% goat serum at 1:400 for 1 hr at room temperature. TBST washes was repeated three times before capturing images at 20X magnification using a Nikon Eclipse 80i microscope and NIS-Elements software and image contrast was performed for the entire image using Adobe photoshop. The display lookup tables are presented in Supplementary Fig. S3.

### Flow cytometry

Jurkat cells nucleofected with PMO for 3 days were incubated with PE fluorophore labeled anti-human ITGA4 antibody (BD Pharmingen, cat. no. 555503) at the recommended concentration, for 25 min on ice before analysis using a Beckman Coulter Gallios flow cytometer. For secondary lymphoid organs (SLOs), cells were resuspended, counted and plated at 4 × 10^6^ per well in round-bottom 96 well plates pre-coated with anti-CD3 antibody (10ug/ml) and cultured for 4 hours in the presence of Golgiblock (Brefeldin A, Invitrogen™) and anti-CD28 (1ug/ml) (BD Bioscience). For brains, cell suspensions were resuspended, counted and all remaining harvested cells were plated in round-bottom 96 well plates coated with 10 μg anti-CD3 antibody and cultured for 4 hours at 37 °C in the presence of Golgiblock and anti-CD28 (1 μg/ml). Cells were then harvested and washed in cold PBS/1% BSA/0.04% azide and Fc receptors were blocked using murine gamma globulin (Rockland), cell surface antigens were stained (CD45, CD11b, CD4, CD8, TCRb, BD Bioscience) and dead cells identified with fixable infra-red viability stain (BD Bioscience). Cells were then fixed and permeablised using the Foxp3 staining kit (eBioscience) and intracellular antigens stained (FoxP3, IL-10, IL-17A, IFNg and GM-CSF, all from BD Biosciences). Following washes, cells were resuspended in cold PBS/0.04% sodium azide and analysed using a BD LSR-Fortessa cytometer. Compensation controls were freshly prepared using UltraComp beads (eBioscience) conjugated to the antibodies used in the study or cells stained only with the cell viability dye. Raw data are provided as Tables S6 and S7.

### Statistics

Two-tailed student t-tests were used for all *in vitro* studies. Two-way ANOVAs with Bonferroni post-tests was performed for clinical scores during the *in vivo* study and one-way ANOVAs for flow cytometry analysis.

## Supplementary information

Supplementary figures and methods

Supplementary tables

## References

[1] Zamecnik PC, Stephenson ML (1978). Inhibition of Rous sarcoma virus replication and cell transformation by a specific oligodeoxynucleotide. Proc. Natl. Acad. Sci. USA.

[2] Stein CA, Castanotto D (2017). FDA-Approved Oligonucleotide Therapies in 2017. Mol. Ther..

[3] FDA grants accelerated approval to first drug for Duchenne muscular dystrophy (press release), https://www.fda.gov/newsevents/newsroom/pressannouncements/ucm521263.htm (2016).

[4] Ottesen EW (2017). ISS-N1 makes the First FDA-approved Drug for Spinal Muscular Atrophy. Transl. Neurosci..

[5] Wilton SD, Fletcher S (2011). RNA splicing manipulation: strategies to modify gene expression for a variety of therapeutic outcomes. Curr. Gene Ther..

[6] Mitroulis I (2015). Leukocyte integrins: role in leukocyte recruitment and as therapeutic targets in inflammatory disease. Pharmacol. Ther..

[7] Hogg N, Laschinger M, Giles K, McDowall A (2003). T-cell integrins: more than just sticking points. J. Cell Sci..

[8] Schwab N, Schneider-Hohendorf T, Wiendl H (2015). Therapeutic uses of anti-alpha4-integrin (anti-VLA-4) antibodies in multiple sclerosis. Int. Immunol..

[9] Mattioli F (2015). Natalizumab Significantly Improves Cognitive Impairment over Three Years in MS: Pattern of Disability Progression and Preliminary MRI Findings. PLoS One.

[10] Schwab N (2017). Natalizumab-associated PML: Challenges with incidence, resulting risk, and risk stratification. Neurology.

[11] Vennegoor A (2013). Clinical relevance of serum natalizumab concentration and anti-natalizumab antibodies in multiple sclerosis. Mult. Scler..

[12] Myers KJ (2005). Antisense oligonucleotide blockade of alpha 4 integrin prevents and reverses clinical symptoms in murine experimental autoimmune encephalomyelitis. J. Neuroimmunol..

[13] Limmroth V (2014). CD49d antisense drug ATL1102 reduces disease activity in patients with relapsing-remitting MS. Neurology.

[14] Flierl U (2015). Phosphorothioate backbone modifications of nucleotide-based drugs are potent platelet activators. J. Exp. Med..

[15] Toonen LJA (2018). Intracerebroventricular Administration of a 2′-O-Methyl Phosphorothioate Antisense Oligonucleotide Results in Activation of the Innate Immune System in Mouse Brain. Nucleic Acid Ther..

[16] Chakravarthy M, Aung-Htut MT, Le BT, Veedu RN (2017). Novel Chemically-modified DNAzyme targeting Integrin alpha-4 RNA transcript as a potential molecule to reduce inflammation in multiple sclerosis. Sci. Rep..

[17] Constantinescu CS, Farooqi N, O’Brien K, Gran B (2011). Experimental autoimmune encephalomyelitis (EAE) as a model for multiple sclerosis (MS). Br. J. Pharmacol..

[18] Desmet FO (2009). Human Splicing Finder: an online bioinformatics tool to predict splicing signals. Nucleic Acids Res..

[19] Mann CJ (2002). Improved antisense oligonucleotide induced exon skipping in the mdx mouse model of muscular dystrophy. J. Gene Med..

[20] Luo YB (2014). Antisense oligonucleotide induction of progerin in human myogenic cells. PLoS One.

[21] Fragall CT (2011). Mismatched single stranded antisense oligonucleotides can induce efficient dystrophin splice switching. BMC Med. Genet..

[22] Chen GL (2017). Modulation of nuclear REST by alternative splicing: a potential therapeutic target for Huntington’s disease. J. Cell Mol. Med..

[23] Morgan JE (1994). Myogenic cell lines derived from transgenic mice carrying a thermolabile T antigen: a model system for the derivation of tissue-specific and mutation-specific cell lines. Dev. Biol..

[24] Labrijn AF (2009). Therapeutic IgG4 antibodies engage in Fab-arm exchange with endogenous human IgG4 *in vivo*. Nat. Biotechnol..

[25] Wollebo HS (2011). Role for tumor necrosis factor-alpha in JC virus reactivation and progressive multifocal leukoencephalopathy. J. Neuroimmunol..

[26] Cannella B, Gaupp S, Tilton RG, Raine CS (2003). Differential efficacy of a synthetic antagonist of VLA-4 during the course of chronic relapsing experimental autoimmune encephalomyelitis. J. Neurosci. Res..

[27] McClorey G (2006). Antisense oligonucleotide-induced exon skipping restores dystrophin expression *in vitro* in a canine model of DMD. Gene Ther..

[28] Flynn, L. L. *et al*. Interaction of modified oligonucleotides with nuclear proteins, formation of novel nuclear structures and sequence-independent effects on RNA processing. Preprint at https://www.biorxiv.org/content/biorxiv/early/2019/01/08/446773.full.pdf (2019).

[29] Adams AM (2007). Antisense oligonucleotide induced exon skipping and the dystrophin gene transcript: cocktails and chemistries. BMC Mol. Biol..

[30] Mendell J, Powers J, Duda P, Eliopoulos H (2016). Clinical safety of eteplirsen, a phosphorodiamidate morpholino oligomer (PMO), in Duchenne muscular dystrophy (DMD) patients amenable to skipping exon 51 of the DMD gene. Neuromuscular Disorders.

[31] Frazier KS (2015). Antisense oligonucleotide therapies: the promise and the challenges from a toxicologic pathologist’s perspective. Toxicol. Pathol..

[32] Pinto-Mariz F (2015). CD49d is a disease progression biomarker and a potential target for immunotherapy in Duchenne muscular dystrophy. Skelet. Muscle.

[33] Sureau A (2001). SC35 autoregulates its expression by promoting splicing events that destabilize its mRNAs. EMBO J..

[34] Wilson GM (1999). Regulation of AUF1 expression via conserved alternatively spliced elements in the 3′ untranslated region. Mol. Cell Biol..

[35] Piva F, Giulietti M, Nocchi L, Principato G (2009). SpliceAid: a database of experimental RNA target motifs bound by splicing proteins in humans. Bioinformatics.

[36] Uotila LM (2014). Specific phosphorylations transmit signals from leukocyte beta2 to beta1 integrins and regulate adhesion. J. Biol. Chem..

[37] Ding Y (2015). Reg3alpha Overexpression Protects Pancreatic beta Cells from Cytokine-Induced Damage and Improves Islet Transplant Outcome. Mol. Med..

[38] Wu Z, Wang H, Fang S, Xu C (2018). Roles of endoplasmic reticulum stress and autophagy on H2O2induced oxidative stress injury in HepG2 cells. Mol. Med. Rep..

[39] Liang CC, Park AY, Guan JL (2007). *In vitro* scratch assay: a convenient and inexpensive method for analysis of cell migration *in vitro*. Nat. Protoc..

[40] Conant CG, Schwartz MA, Ionescu-Zanetti C (2010). Well plate-coupled microfluidic devices designed for facile image-based cell adhesion and transmigration assays. J. Biomol. Screen..

[41] Ghavampour S, Lange C, Bottino C, Gerke V (2013). Transcriptional profiling of human monocytes identifies the inhibitory receptor CD300a as regulator of transendothelial migration. Plos One.

[42] Bancroft, J. D., Layton, C. & Suvarna, K. S. *Bancroft’s Theory and Practice of Histological Techniques* 7 th edn, (Churchill Livingstone Elsevier- (Oxford) 2013).

