# Supplementary material for: Reduction of integrin alpha 4 activity through splice modulating antisense oligonucleotides
Source: Sci Rep. 2019 Sep 10;9:12994. doi: 10.1038/s41598-019-49385-6 (PMC6736852; doi:10.1038/s41598-019-49385-6)
Supplement: Supplementary file 1 — Supplementary figures and methods [file 41598_2019_49385_MOESM1_ESM.pdf]

Reduction of integrin alpha 4 activity through splice modulating antisense oligonucleotides

May T. Aung-Htut[1,2], Iain Comerford[3], Russell Johnsen[1,2], Kerrie Foyle[3], Sue Fletcher[1,2], Steve D Wilton[1,2*]

[1]Centre for Molecular Medicine and Innovative Therapeutics, Murdoch University, Perth, Western Australia.

[2]Perron Institute for Neurological and Translational Science, University of Western Australia, Nedlands, Western Australia.

[3]Chemokine Biology Lab, School of Biological Sciences, University of Adelaide, Adelaide, South Australia.

*Correspondence should be addressed to S.D.W. (s.wilton@murdoch.edu.au)

Supplementary figures

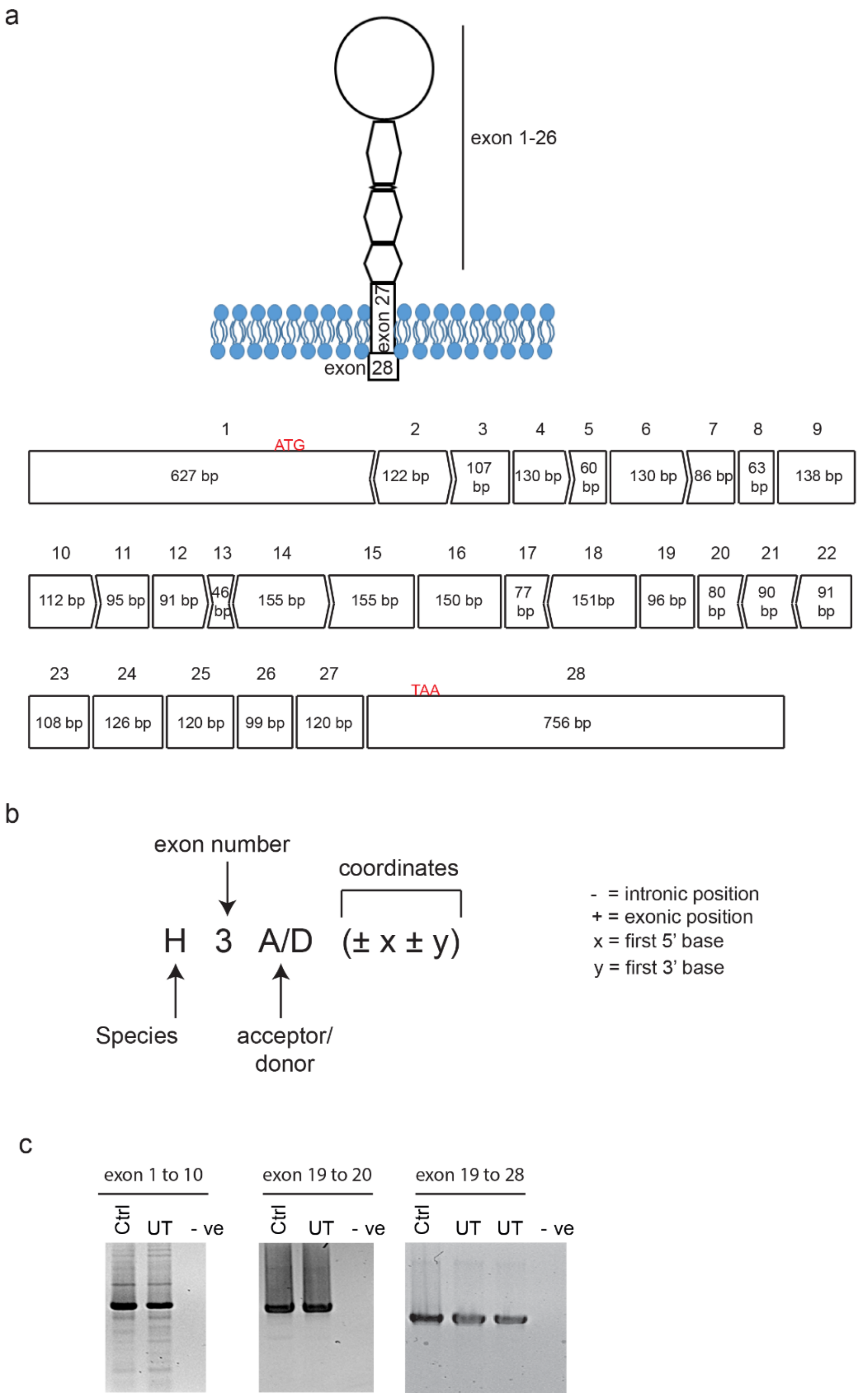

Figure S1. (a) A schematic of integrin alpha 4 (ITGA4) receptor and the reading frame of the *ITGA4* transcript. Exons are presented as boxes. Exon numbers are shown above the boxes and the size of each exon in base pair (bp) is also shown. The start and stop codons are in red. (b) Antisense oligonucleotide nomenclature. (c) RT-PCR products of *ITGA4* transcript amplified from normal human fibroblasts untreated (UT) and treated with control AO (Ctrl) at 100 nM.

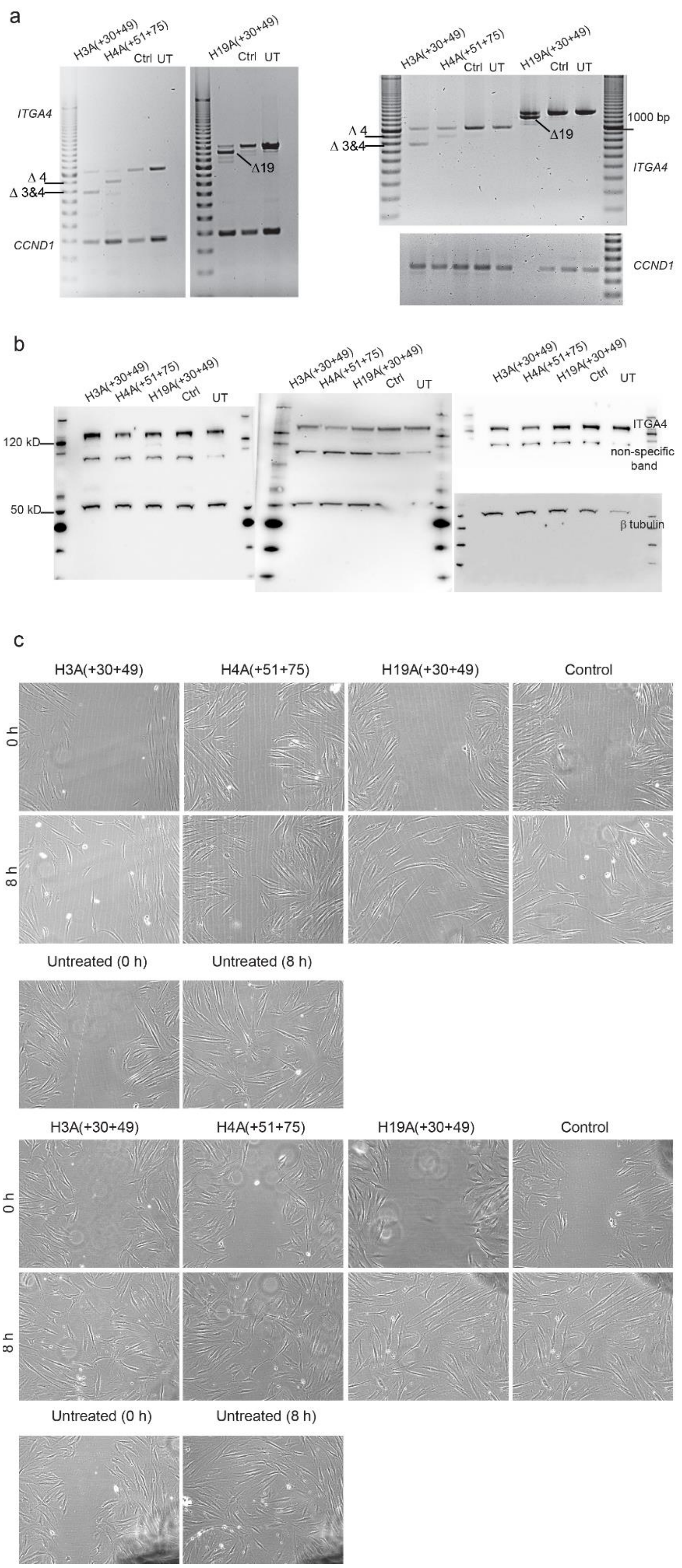

Figure S2. Biological replicates for the analysis of *ITGA4* transcript, ITGA4 protein expression and activity of healthy dermal fibroblasts treatment with the top three exon skipping AOs targeting exon 3,

4 or 19 for 48 hr. (a) Gel fractionation of RT-PCR products of *ITGA4* amplicons amplified from healthy dermal fibroblasts transfected with the 2OMe PS AOs at 100 nM for 48 hr. Ctrl: control AO, UT: untreated. *CCND1* transcript encoding cyclin D protein was used as a loading control. (b) Western analysis of ITGA4 protein expression and (c) analysis of fibroblast migration using an established wound healing assay, using the treated and untreated healthy dermal fibroblasts from (a). Beta tubulin was used as a reference protein for western analysis.

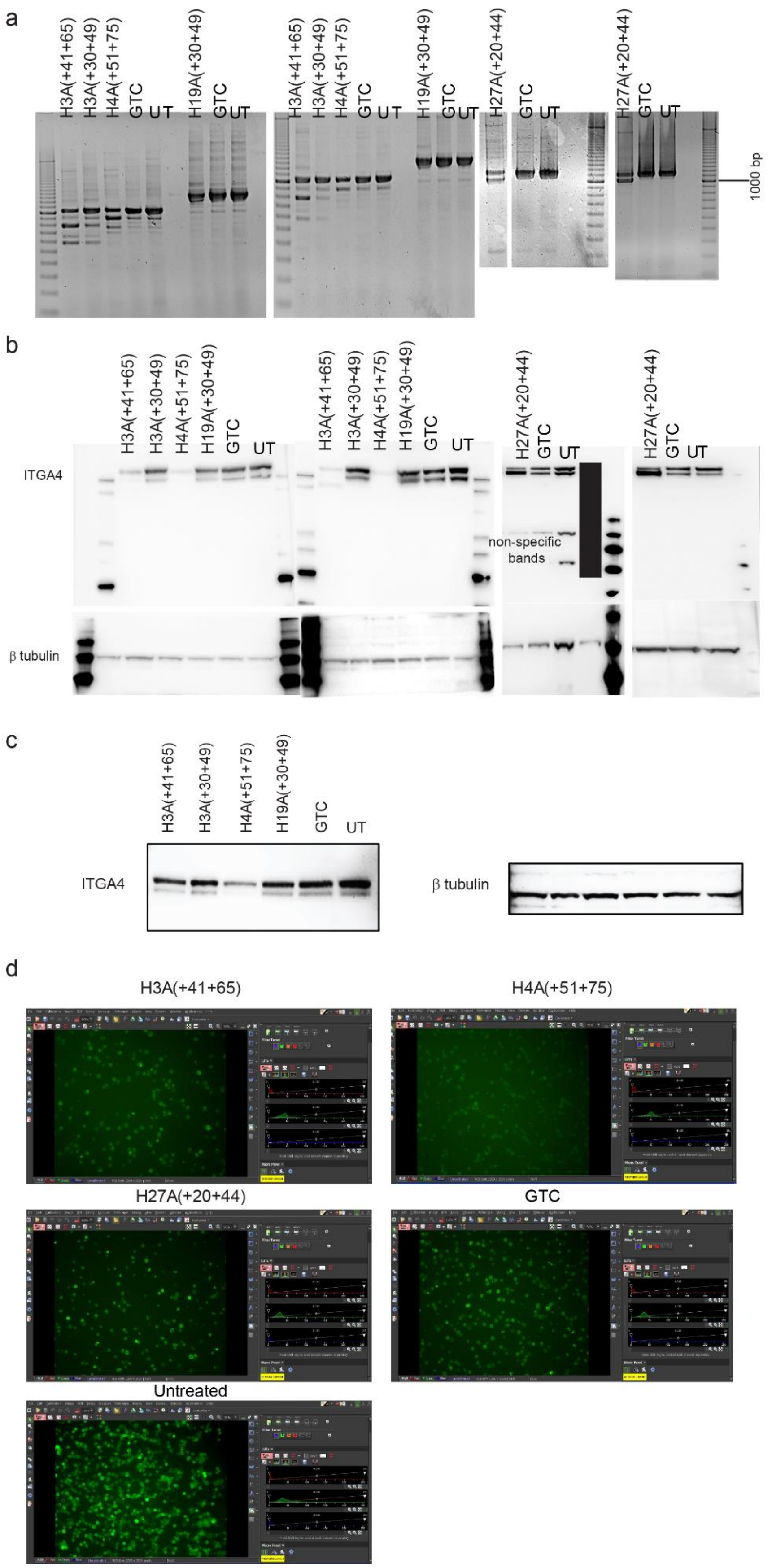

Figure S3. Biological replicates for the analysis of *ITGA4* transcript, ITGA4 protein expression and activity of Jurkat cells treated with indicated PMOs for three days. (a) Gel fractionation of RT-PCR products of the *ITGA4* transcript and (b) western blot analysis of ITGA4 protein from Jurkat cells

nucleofected with PMOs, as indicated above the gel, at 50 µM for three days and (c) 6 days. Beta tubulin was used as a reference protein. (d) The display lookup table (LUT) for images taken for ITGA4 protein in Figure 3d. GTC: Gene Tools control AO, UT: untreated.

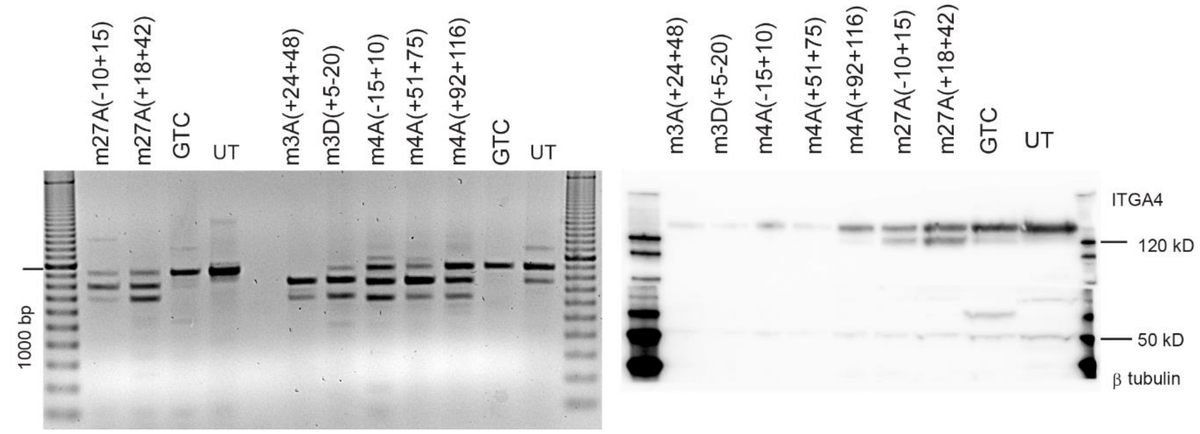

Figure S4. Biological replicates for Gel fractionation of RT-PCR products of murine *Itga4* transcript (left) and western analysis of ITGA4 protein (right) from primary murine splenocytes nucleofected with PMOs, as shown above the gel, at 50 µM for 48 h.

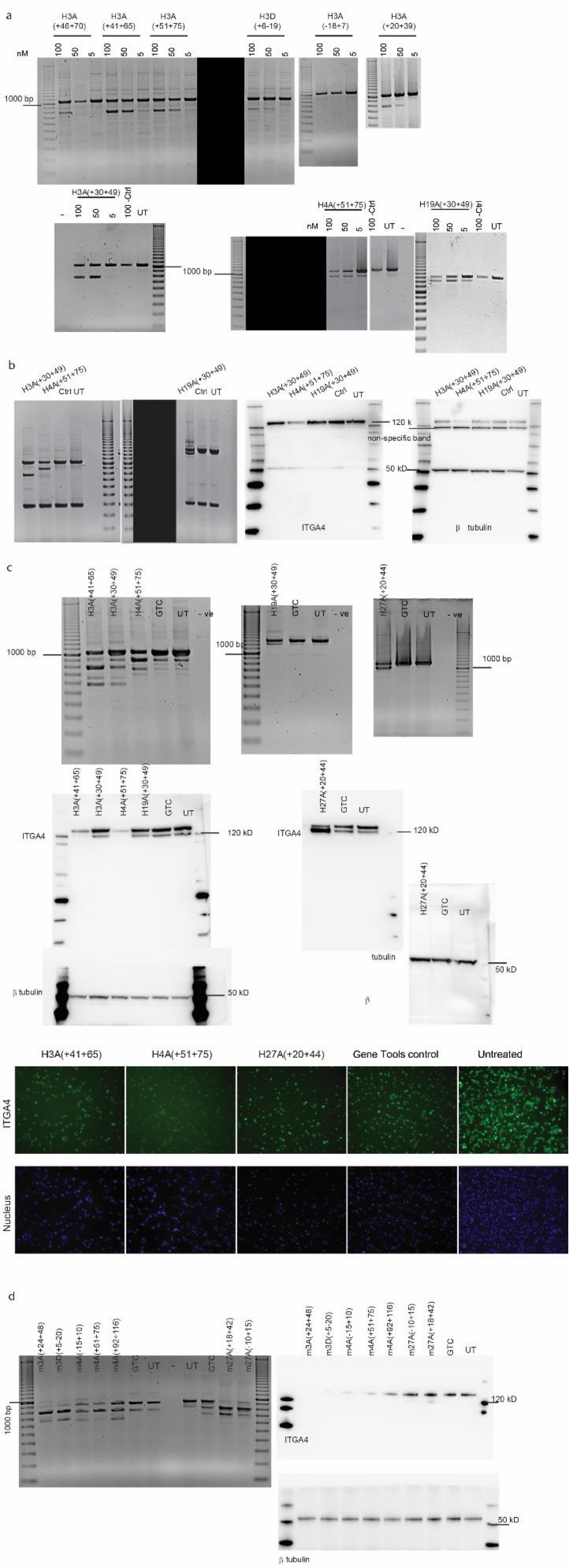

Figure S5. The full-length gels and blots for (a) Figure 1b and c (b) Figure 2a and b (c) Figure 3a,b and c (d) Figure 4 a and b.

Supplementary methods

Transfection/nucleofection

Cells (15-17,000 in 24 wells) were seeded one day before transfecting with 2OMe PS AOs using Lipofectin® transfection reagent (Lipofectin:oligo; 2:1) according to the manufacturer's protocol. Transfected cells were incubated for 24 hours before extracting RNA using Trizol, according to manufacturer's guidelines. Transfections were performed in T25 flasks for protein analysis and the cells harvested at 48 hours. Jurkat cells were supplied by the European Collection of Cell Cultures (ECACC; Salisbury, United Kingdom) as catalogue number 88042803, and was purchased from CellBank Australia (Westmead, NSW, Australia) and maintained in 10% FBS, RPMI-1640. The PMOs were nucleofected into Jurkat cells using the P3 Primary Cell 4D-nucleofactor X Kit (Lonza). Approximately, 500,000 cells were nucleofected with the PMO at 50 µM using program CL-120 and incubated in 5% FCS, RPMI-1640 for 3 days for RNA analysis, or 3 or 6 days for western.

Western blotting

Cells were lysed in buffer (15% SDS, 50 mM Tris HCl pH 6.8, 10% glycerol, 1 mM dithiothreitol), supplemented with complete protease inhibitor cocktails and SDS-PAGE electrophoresis was performed before transferring to Pall FluoroTrans® membranes and incubation with rabbit anti-ITGA4 antibody (Cell Signaling Technology, cat. no. 4600)[36] at 1:1000 dilution and rabbit polyclonal anti-β-tubulin (Invitrogen™, cat. no. PA1-41331)[37] at 1:1000 dilution overnight at 4°C. Polyclonal goat anti-rabbit immunoglobulins/HRP (Dako, cat. no P0448)[38] at a dilution of 1:10,000 and Luminata Crescendo western HRP substrate were used for immunodetection. The blots were exposed for a serial scan of 30s using Fusion FX system (Vilber Lourmat, Marne-la-Vallée, France). Image processing was performed for the entire image. Quantification was performed using Image J (Rasband, W. S., ImageJ, U. S. National Institutes of Health, Bethesda, Maryland, USA).

Cell adhesion assay

The assay was performed as described[40] with slight modifications. Ninety-six well microplates were coated with fibronectin (3 µg/well in PBS), laminin (0.75 µg/ well in PBS) and recombinant Human VCAM-1 (0.5 µg / well in PBS) overnight at room temperature and blocked with 1% BSA for 60 min at 37°C. The untreated and transfected fibroblasts were harvested, washed twice in PBS and labelled with 2 µM calcein AM fluorescent dye for 30 min in serum free (SF) DMEM. After two washes with SF-DMEM, the cells were resuspended in 0.6 -1 ml 10% FCS, DMEM. Cell suspension (50 µl) was added to each well and incubated at 37°C for 30-40 min. The microplates were washed four times with PBS, and the remaining adherent cells were measured using a Beckman Coulter DTX-880 Multimode Detector plate reader with excitation and emission wavelengths of 488 nm and 512 nm, respectively. The fluorescent signals from total cells were analysed in a separate microplate, omitting the wash steps. Background signals were subtracted from all samples and the percentages of adhered cells were calculated. The results were normalised to the sample treated with the control AO.

Jurkat cell migration assay

The assay was performed as described[41] with slight modifications. The upper-sides of transwell migration inserts (Polyester membrane, 3 µm pore size, 6.5 mm diameter) were coated with 50 µl of 0.5 µg/µl recombinant human VCAM-1 overnight at room temperature and pre-equilibrated with RPMI-1640 for 1-2 h at 37°C. Jurkat cells were nucleofected with PMO and incubated for 2 days before resuspending in 100 µl of SF-PRMI-1640 media and addition to the upper compartment of the insert. Media (600 µl of 10% FCS, RPMI-1640) was added to the lower compartment. The cells were allowed to migrate from the upper to lower chambers for 5h at 37°C. Cells from both chambers were collected and incubated with 2 µM calcein AM fluorescent dye for 30 min before measuring fluorescent signals as described above. The percentages of cells that migrated to the lower chambers were calculated and normalised to the signals generated by the cells treated with GTC PMO.

In vivo validation in EAE mouse model

In vivo study to validate the efficacy of PPMO was performed at the University of Adelaide (**University of Adelaide ethics approval S-2017-092**). All methods were performed in accordance with the relevant guidelines and regulations. Sixty female C57Bl/6J mice aged 7-12 weeks were obtained from the Animal Resources Centre in Perth. Mice were housed in individually ventilated cages with access to food and water *ad libitum*. Mice were immunized with 200 µg of MOG35-55 (BioNovus Life Science) peptide emulsified 1:1 in complete Freund's adjuvant (Sigma) in a total volume of 100µl per mouse, subcutaneously in the hind flank. On day 0 and on day 2, mice received 300 ng of pertussis toxin (Sigma) in a total volume of 250 µl PBS intravenously. Mice were split into two cohorts of 30 mice immunized one day apart. Mice were weighed and clinical disease assessed daily in a blinded manner according to the following criteria as described in Table S5. Eight of the 60 mice immunized were not assigned to experimental groups on day 12 because they had either already met euthanasia criteria or had developed a form of atypical EAE characterized by ataxia and loss of co-ordination that cannot be adequately scored using the standard EAE clinical disease assessment described above. Mice that were found dead or culled due to meeting animal welfare euthanasia criteria (>20% weight loss or excessive clinical disease) were scored at the same clinical score they had last attained for the remainder of the study.

All PPMOs were diluted to 53mg/ml in sterile MQ water and stored at 4°C. On day 12 post-immunisation (when the mean clinical disease score of the whole cohort was greater than 1), mice were split into one of 6 groups at random making sure an equivalent distribution of disease scores across the groups. Mice were treated intraperitoneally with 10mg/kg of PPMO in PBS (i.e. 200µl of a 1mg/ml dilution of PMO stock in sterile PBS per 20g of body weight) on day 12 and then every 48 hr thereafter. For rat anti-mouse ITGA4 antibody (Cat. 1520-14, Assay Matrix Pty Ltd) treatment as a positive control, mice were given 25 mg/kg on day 12 and 5 mg/kg every 48 hr thereafter. Equivalent volumes of PBS were administered

intraperitoneally to control mice at the same time. PPMO and antibody dilutions were freshly prepared from stocks in sterile PBS.

Histology

The lumbar part of spinal cords were embedded in OCT and snap frozen in blocks using liquid nitrogen then stored at -80°C. Six micron fresh frozen sections of OCT embedded mouse spinal cord samples were cut on a cryostat (Leica CM1900), and mounted on microscope slides. Sections were stained using a standard haematoxylin and eosin stain[42] for microscopic identification of inflammation using a Nikon Eclipse 80i microscope at 10X magnification. Using NIH ImageJ software, the total area of each tissue section and areas of inflammation, with the presence of mononuclear inflammatory cells within the tissue were selected and measured. For each microscopic image analysed, measurements of areas of inflammation were presented as percent of total area of the field of views analysed. The operator was blinded for analysis.

I*mmunolabeling*

For immunolabeling of ITGA4 protein, Jurkat cells grown on poly D lysine coated coverslips were fixed in ice-cold Acetone:MetOH (1:1) for 5 min before incubation with primary antibody (Cell Signaling Technology, cat. no. 8440) diluted in TBST with 1% goat serum at 1:200 for 1 h at room temperature. The coverslips were then washed three times with TBST before incubation with Alexa 488 labelled goat anti-rabbit secondary antibody (ThermoFisher cat. no. A27034) diluted in TBST with 1% goat serum at 1:400 for 1 hr at room temperature. Washing with TBST was repeated three times before mounting on to glass slides using ProLong Gold antifade mountant. Images were captured at 20X magnification using a Nikon Eclipse 80i microscope and processing was performed for the entire image.

Flow cytometry

Jurkat cells were collected 3 days after nucleofection and washed twice with cold PBS before incubating with PE fluorophore labeled anti-human ITGA4 antibody (BD Pharmingen,

cat. no. 555503) at the recommended concentration, for 25 min on ice. Cells were washed once with cold PBS and analysed using a Beckman Coulter Gallios flow cytometer. For secondary lymphoid organs (SLOs), cell suspensions were resuspended in complete (10% FCS) Iscove's Modified Dulbecco's Medium (IMDM), counted and plated at $4x10^6$ per well in round-bottom 96 well plates pre-coated with anti-CD3 antibody (10ug/ml) and cultured for 4 hours in the presence of Golgiblock (Brefeldin A, Invitrogen™) and anti-CD28 (1ug/ml) (BD Bioscience). For brains, cell suspensions were resuspended in complete (10% FCS) IMDM, counted and all remaining harvested cells were plated in round-bottom 96 well plates coated with 10µg anti-CD3 antibody and cultured for 4 hours at 37°C in the presence of Golgiblock and anti-CD28 (1µg/ml). Cells were then harvested and washed in cold PBS/1% BSA/0.04% azide and Fc receptors were blocked using murine gamma globulin (Rockland), cell surface antigens were stained (CD45, CD11b, CD4, CD8, TCRb, BD Bioscience) and dead cells identified with fixable infra-red viability stain (BD Bioscience). Cells were then fixed and permeablised using the Foxp3 staining kit (eBioscience) and intracellular antigens stained (FoxP3, IL-10, IL-17A, IFNg and GM-CSF, all from BD Biosciences). Following washes, cells were resuspended in cold PBS/0.04% sodium azide and analysed using a BD LSR-Fortessa cytometer. Compensation controls were freshly prepared using UltraComp beads (eBioscience) conjugated to the antibodies used in the study or cells stained only with the cell viability dye. Fluorescence minus one control for each channel were also prepared and acquired. For each 200 µl sample around 180 µl was recorded.
